# *N*-[2-(2-Methoxyphenyl)benzylidene]-*tert*-butyl­amine *N*-oxide

**DOI:** 10.1107/S1600536808014529

**Published:** 2008-05-17

**Authors:** Jin-Long Wu, Yu Liao, Shan-Lin Liu

**Affiliations:** a Laboratory of Asymmetric Catalysis and Synthesis, Department of Chemistry, Zhejiang University, Hangzhou, Zhejiang 310027, People’s Republic of China; b Department of Chemistry, Zhejiang University, Hangzhou, Zhejiang 310027, People’s Republic of China

## Abstract

In the mol­ecule of the title compound, C_18_H_21_NO_2_, the two benzene rings are oriented at a dihedral angle of 58.19 (3)°. Intra­molecular C—H⋯O hydrogen bonds result in the formation of one six- and one five-membered ring, which adopt twist and envelope conformations, respectively. In the crystal structure, C—H⋯O hydrogen bonds link the mol­ecules.

## Related literature

For general background, see: Hamburger & McCay (1989 ▶); Jotti *et al.* (1992 ▶); Murphy *et al.* (2003 ▶); Green *et al.* (2003 ▶); Durand *et al.* (2007 ▶); Hay *et al.* (2005 ▶). For related literature, see: Fevig *et al.* (1996 ▶).
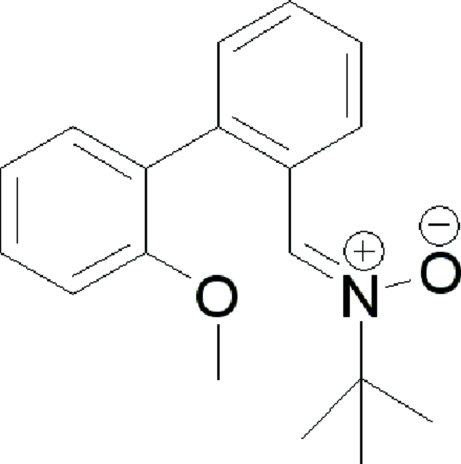


## Experimental

### 

#### Crystal data

C_18_H_21_NO_2_
*M* *_r_* = 283.37
Monoclinic, 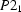

*a* = 10.2526 (15) Å
*b* = 8.5576 (13) Å
*c* = 10.3333 (16) Å
β = 115.742 (3)°
*V* = 816.6 (2) Å^3^
*Z* = 2
Mo *K*α radiation
μ = 0.07 mm^−1^
*T* = 296 (1) K
0.30 × 0.28 × 0.09 mm

#### Data collection

Rigaku R-AXIS RAPID-S diffractometer
Absorption correction: multi-scan (*ABSCOR*; Higashi, 1995 ▶) *T*_min_ = 0.968, *T*_max_ = 0.993
7869 measured reflections
1981 independent reflections
967 reflections with *F* ^2^ > 2σ(*F* ^2^)
*R*_int_ = 0.035

#### Refinement

*R*[*F* ^2^ > 2σ(*F* ^2^)] = 0.034
*wR*(*F* ^2^) = 0.058
*S* = 1.00
1981 reflections
191 parameters
H-atom parameters constrained
Δρ_max_ = 0.23 e Å^−3^
Δρ_min_ = −0.21 e Å^−3^

### 

Data collection: *PROCESS-AUTO* (Rigaku, 1998 ▶); cell refinement: *PROCESS-AUTO*; data reduction: *CrystalStructure* (Rigaku/MSC, 2004 ▶) and Larson (1970 ▶); program(s) used to solve structure: *SIR97* (Altomare *et al.*, 1999 ▶); program(s) used to refine structure: *CRYSTALS* (Betteridge *et al.*, 2003 ▶); molecular graphics: *ORTEP-3 for Windows* (Farrugia, 1997 ▶); software used to prepare material for publication: *CrystalStructure*.

## Supplementary Material

Crystal structure: contains datablocks global, I. DOI: 10.1107/S1600536808014529/hk2462sup1.cif

Structure factors: contains datablocks I. DOI: 10.1107/S1600536808014529/hk2462Isup2.hkl

Additional supplementary materials:  crystallographic information; 3D view; checkCIF report

## Figures and Tables

**Table 1:** Hydrogen-bond geometry (Å, °)

*D*—H⋯*A*	*D*—H	H⋯*A*	*D*⋯*A*	*D*—H⋯*A*
C3—H3⋯O1	0.93	2.26	2.806 (3)	117
C17—H171⋯O1	0.96	2.41	2.791 (3)	104
C18—H181⋯O1^i^	0.96	2.50	3.280 (3)	139

Symmetry code: (i) 
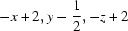
.

**Fig. 1.**
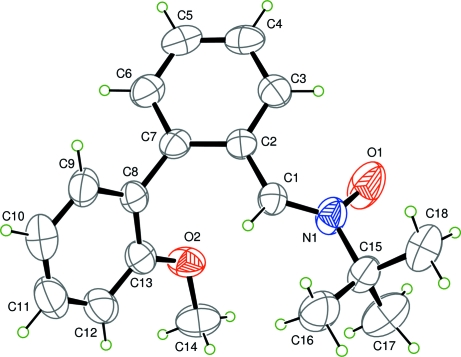
The molecular structure of the title molecule, with the atom-numbering scheme. Displacement ellipsoids are drawn at the 40% probability level.

**Fig. 2.**
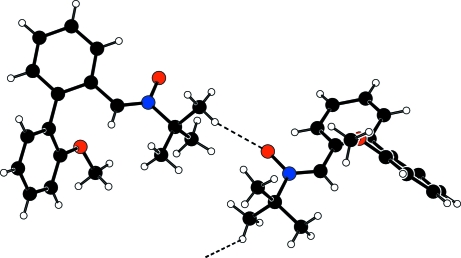
A partial packing diagram of (I). Hydrogen bonds are shown as dashed lines.

## supplementary crystallographic information

### Comment

*N*-*tert*-Butyl-α-phenylnitrone (PBN) and its derivatives have been widely used as spin trapping agents in biology for detecting active free radicals such as oxygen- and carbon-centered radicals (Hamburger & McCay, 1989; Jotti *et al.*, 1992; Murphy *et al.*, 2003) and as antioxidant for treating age-related diseases (Green *et al.*, 2003). The amphiphilic spin traps have been developed for potential use as therapeutic antioxidants (Durand *et al.*, 2007), while the lipophilic spin traps are targeted for interception of radicals within non-aqueous phases and detection of radicals in complex biphasic biological systems (Hay *et al.*, 2005). To explore the effect of the substituent attached to the benzene ring of PBN on the spin trapping ability, we have designed and synthesized a number of novel lipophilic spin traps derived from PBN, to which an additional aromatic group is attached.

In the molecule of (I), (Fig. 1), rings A (C2-C7) and B (C8-C13) are, of course, planar, and they are oriented at a dihedral angle of 58.19 (3)°. Unlike the *N*-*tert*-Butyl-α-phenylnitrone (Fevig *et al.*, 1996), atoms C1, N1 and O1 is not coplanar with ring A. Intramolecular C-H···O hydrogen bonds result in the formation of one six- and one five-membered rings C (C1-C3/O1/N1/H3) and D (O1/N1/C15/C17/H171). Ring C adopts twisted conformation having total puckering amplitude, Q_T_, of 0.443 (3) Å, while ring D has envelope conformation, with C15 atom displaced by -0.663 (3) Å from the plane of the other ring atoms.

In the crystal structure, intermolecular C-H···O hydrogen bonds link the molecules (Fig. 2), in which they may be effective in the stabilization of the structure.

### Experimental

For the preparation of the title compound, activated zinc powder (195.0 mg, 3.0 mmol) was added to a solution of 2-(2-methoxyphenyl)benzaldehyde (212.0 mg, 1.0 mmol) and 2-methyl-2-nitropropane (309.0 mg, 3.0 mmol) in ethanol (95%, 15 ml) at 283 K. Glacial acetic acid (0.35 ml, 6.0 mmol) was added dropwise to the resultant suspension in 1 h with stirring. After stirring at room temperature for 12 h, the reaction mixture was kept for another 48 h in a refrigerator. It was filtered to remove the solid materials and the filtrate was concentrated under reduced pressure. The residue was purified by column chromatography on silica gel with ethylacetate and hexane (1:3) as the eluent to give the title compound (yield; 122.0 mg, 43%, m.p. 351-353 K). 1H NMR (400 MHz, CDCl_3_) δ: 9.43 (d, J = 8.8 Hz, 1H), 7.43-7.37 (m, 3H), 7.31 (s, 1H), 7.28-7.26 (m,1H), 7.17 (d, J = 6.4 Hz, 1H), 7.04 (t, J = 7.2 Hz, 1H), 6.98 (d, J = 8.0 Hz, 1H), 3.72 (s, 3H), 1.42 (s, 9H); 13 C NMR (100 MHz, CDCl_3_) δ: 156.2, 138.9, 131.5, 130.0, 129.5, 129.4, 129.2, 128.8 (2), 127.5, 127.4, 120.7 (2), 110.5, 70.6, 55.3, 28.0 (3); MS (ESI) m/*z* 284 (*M* + H). Anal. Calcd for C_18_H_21_NO_2_: C, 76.29; H, 7.47; N, 4.94. Found: C, 76.40; H, 7.45; N, 4.67. Single crystals of (I) suitable for X-ray analysis were grown in ethylacetate and hexane.

### Refinement

H atoms were positioned geometrically, with C-H = 0.93 and 0.96 Å for aromatic and methyl H, respectively, and constrained to ride on their parent atoms with U_iso_(H) = 1.2U_eq_(C).

### Crystal data

**Table:**

C_18_H_21_NO_2_	*F*_000_ = 304.00
*M**_r_* = 283.37	*D*_x_ = 1.152 Mg m^−^^3^
Monoclinic, *P*2_1_	Mo *K*α radiation λ = 0.71075 Å
Hall symbol: P 2yb	Cell parameters from 4871 reflections
*a* = 10.2526 (15) Å	θ = 3.2–27.4º
*b* = 8.5576 (13) Å	µ = 0.08 mm^−^^1^
*c* = 10.3333 (16) Å	*T* = 296 (1) K
β = 115.742 (3)º	Platelet, colorless
*V* = 816.6 (2) Å^3^	0.30 × 0.28 × 0.09 mm
*Z* = 2	

### Data collection

**Table:**

Rigaku R-AXIS RAPID-S diffractometer	967 reflections with *F*^2^ > 2σ(*F*^2^)
Detector resolution: 10.00 pixels mm^-1^	*R*_int_ = 0.035
ω scans	θ_max_ = 27.4º
Absorption correction: multi-scan(ABSCOR; Higashi, 1995)	*h* = −13→13
*T*_min_ = 0.968, *T*_max_ = 0.993	*k* = −11→10
7869 measured reflections	*l* = −13→13
1981 independent reflections	

### Refinement

**Table:**

Refinement on *F*^2^	*w* = 1/[1.0600σ(*F*_o_^2^)]/(4*F*_o_^2^)
*R*[*F*^2^ > 2σ(*F*^2^)] = 0.034	(Δ/σ)_max_ < 0.001
*wR*(*F*^2^) = 0.058	Δρ_max_ = 0.23 e Å^−^^3^
*S* = 1.00	Δρ_min_ = −0.21 e Å^−^^3^
1981 reflections	Extinction correction: Larson (1970)
191 parameters	Extinction coefficient: 287 (12)
H-atom parameters constrained	

### Special details

**Table:**

Geometry. ENTER SPECIAL DETAILS OF THE MOLECULAR GEOMETRY
Refinement. Refinement using all reflections. The weighted *R*-factor (*wR*) and goodness of fit (*S*) are based on *F*^2^. *R*-factor (gt) are based on *F*. The threshold expression of *F*^2^ > 2.0 σ(*F*^2^) is used only for calculating *R*-factor (gt).

### Fractional atomic coordinates and isotropic or equivalent isotropic displacement parameters (Å^2^)

**Table:**

	*x*	*y*	*z*	*U*_iso_*/*U*_eq_	
O1	0.9021 (2)	0.3664 (2)	0.8067 (2)	0.1211 (8)	
O2	0.89199 (16)	0.2119 (2)	0.40803 (18)	0.0749 (6)	
N1	0.8516 (2)	0.2377 (2)	0.7378 (2)	0.0699 (7)	
C1	0.7579 (2)	0.2359 (3)	0.6043 (2)	0.0539 (7)	
C2	0.6914 (2)	0.3714 (3)	0.5161 (2)	0.0519 (7)	
C3	0.6741 (2)	0.5115 (3)	0.5752 (2)	0.0657 (9)	
C4	0.6018 (2)	0.6370 (3)	0.4891 (3)	0.0749 (9)	
C5	0.5479 (2)	0.6233 (3)	0.3431 (3)	0.0794 (10)	
C6	0.5634 (2)	0.4858 (3)	0.2821 (2)	0.0706 (9)	
C7	0.6339 (2)	0.3570 (3)	0.3658 (2)	0.0552 (8)	
C8	0.6389 (2)	0.2103 (3)	0.2930 (2)	0.0569 (8)	
C9	0.5111 (2)	0.1408 (3)	0.1954 (2)	0.0729 (9)	
C10	0.5107 (3)	0.0054 (4)	0.1245 (2)	0.0890 (11)	
C11	0.6400 (3)	−0.0637 (3)	0.1491 (2)	0.0910 (11)	
C12	0.7694 (3)	0.0020 (3)	0.2437 (2)	0.0775 (10)	
C13	0.7690 (2)	0.1373 (3)	0.3151 (2)	0.0644 (9)	
C14	1.0274 (2)	0.1362 (3)	0.4454 (3)	0.0978 (11)	
C15	0.9129 (2)	0.0920 (3)	0.8265 (2)	0.0704 (9)	
C16	0.8509 (3)	−0.0534 (3)	0.7407 (3)	0.1022 (12)	
C17	1.0746 (2)	0.0986 (4)	0.8838 (3)	0.1160 (12)	
C18	0.8694 (2)	0.1013 (4)	0.9492 (2)	0.1048 (11)	
H1	0.7307	0.1385	0.5610	0.065*	
H3	0.7118	0.5211	0.6745	0.079*	
H4	0.5900	0.7294	0.5302	0.090*	
H5	0.5004	0.7074	0.2847	0.095*	
H6	0.5260	0.4787	0.1826	0.085*	
H9	0.4232	0.1878	0.1778	0.087*	
H10	0.4238	−0.0390	0.0605	0.107*	
H11	0.6405	−0.1558	0.1016	0.109*	
H12	0.8566	−0.0452	0.2591	0.093*	
H141	1.0270	0.0361	0.4873	0.117*	
H142	1.0425	0.1222	0.3608	0.117*	
H143	1.1040	0.1992	0.5135	0.117*	
H161	0.8724	−0.0552	0.6590	0.123*	
H162	0.7479	−0.0546	0.7085	0.123*	
H163	0.8929	−0.1435	0.7995	0.123*	
H171	1.1086	0.1991	0.9263	0.139*	
H172	1.1173	0.0187	0.9550	0.139*	
H173	1.1015	0.0822	0.8066	0.139*	
H181	0.8892	0.0033	0.9993	0.126*	
H182	0.7678	0.1240	0.9117	0.126*	
H183	0.9237	0.1827	1.0144	0.126*	

### Atomic displacement parameters (Å^2^)

**Table:**

	*U*^11^	*U*^22^	*U*^33^	*U*^12^	*U*^13^	*U*^23^
O1	0.1395 (18)	0.0722 (15)	0.0812 (15)	−0.0167 (15)	−0.0179 (12)	−0.0059 (13)
O2	0.0562 (9)	0.0805 (13)	0.0934 (13)	0.0110 (10)	0.0374 (8)	−0.0032 (12)
N1	0.0728 (13)	0.0637 (16)	0.0555 (14)	−0.0094 (14)	0.0113 (11)	−0.0029 (14)
C1	0.0509 (11)	0.0599 (18)	0.0493 (15)	−0.0037 (15)	0.0203 (11)	−0.0017 (15)
C2	0.0405 (12)	0.0608 (18)	0.0561 (16)	−0.0024 (12)	0.0227 (11)	−0.0011 (15)
C3	0.0477 (14)	0.078 (2)	0.0703 (19)	−0.0052 (14)	0.0250 (13)	−0.0124 (18)
C4	0.0504 (14)	0.068 (2)	0.103 (2)	0.0064 (15)	0.0308 (14)	−0.0027 (19)
C5	0.0627 (16)	0.076 (2)	0.097 (2)	0.0188 (17)	0.0328 (16)	0.021 (2)
C6	0.0618 (16)	0.082 (2)	0.069 (2)	0.0091 (15)	0.0299 (14)	0.0130 (19)
C7	0.0431 (13)	0.0695 (19)	0.0572 (17)	0.0068 (14)	0.0257 (11)	0.0083 (17)
C8	0.0621 (14)	0.0682 (19)	0.0450 (14)	0.0043 (16)	0.0274 (12)	0.0012 (15)
C9	0.0699 (17)	0.094 (2)	0.0497 (16)	0.0017 (16)	0.0212 (13)	−0.0003 (17)
C10	0.102 (2)	0.103 (2)	0.0586 (19)	−0.015 (2)	0.0314 (17)	−0.012 (2)
C11	0.126 (2)	0.086 (2)	0.067 (2)	−0.004 (2)	0.0474 (19)	−0.0130 (18)
C12	0.097 (2)	0.078 (2)	0.069 (2)	0.0104 (17)	0.0472 (17)	0.0009 (18)
C13	0.0699 (16)	0.075 (2)	0.0579 (17)	0.0022 (16)	0.0366 (13)	0.0008 (16)
C14	0.0666 (15)	0.105 (2)	0.130 (2)	0.0198 (18)	0.0496 (15)	0.008 (2)
C15	0.0706 (17)	0.069 (2)	0.0573 (18)	0.0000 (17)	0.0146 (13)	0.0103 (17)
C16	0.129 (2)	0.066 (2)	0.087 (2)	0.008 (2)	0.0230 (19)	0.0098 (19)
C17	0.0771 (17)	0.121 (2)	0.124 (2)	0.003 (2)	0.0196 (16)	0.033 (2)
C18	0.113 (2)	0.120 (2)	0.0668 (19)	0.010 (2)	0.0254 (17)	0.025 (2)

### Geometric parameters (Å, °)

**Table:**

O1—N1	1.291 (3)	C1—H1	0.930
O2—C13	1.366 (2)	C3—H3	0.930
O2—C14	1.425 (3)	C4—H4	0.930
N1—C1	1.293 (2)	C5—H5	0.930
N1—C15	1.514 (3)	C6—H6	0.930
C1—C2	1.450 (3)	C9—H9	0.930
C2—C3	1.391 (4)	C10—H10	0.930
C2—C7	1.407 (3)	C11—H11	0.930
C3—C4	1.386 (3)	C12—H12	0.930
C4—C5	1.368 (4)	C14—H141	0.960
C5—C6	1.376 (4)	C14—H142	0.960
C6—C7	1.394 (3)	C14—H143	0.960
C7—C8	1.476 (3)	C16—H161	0.960
C8—C9	1.393 (3)	C16—H162	0.960
C8—C13	1.399 (3)	C16—H163	0.960
C9—C10	1.370 (4)	C17—H171	0.960
C10—C11	1.371 (5)	C17—H172	0.960
C11—C12	1.381 (3)	C17—H173	0.960
C12—C13	1.373 (4)	C18—H181	0.960
C15—C16	1.499 (3)	C18—H182	0.960
C15—C17	1.500 (3)	C18—H183	0.960
C15—C18	1.518 (4)		
			
C13—O2—C14	118.1 (2)	C4—C5—H5	119.8
O1—N1—C1	122.2 (2)	C6—C5—H5	119.8
O1—N1—C15	114.02 (18)	C5—C6—H6	119.2
C1—N1—C15	123.8 (2)	C7—C6—H6	119.2
N1—C1—C2	126.1 (2)	C8—C9—H9	118.9
C1—C2—C3	121.9 (2)	C10—C9—H9	118.9
C1—C2—C7	118.8 (2)	C9—C10—H10	120.4
C3—C2—C7	119.2 (2)	C11—C10—H10	120.4
C2—C3—C4	121.4 (2)	C10—C11—H11	119.7
C3—C4—C5	119.2 (2)	C12—C11—H11	119.7
C4—C5—C6	120.5 (2)	C11—C12—H12	120.1
C5—C6—C7	121.6 (2)	C13—C12—H12	120.1
C2—C7—C6	118.1 (2)	O2—C14—H141	109.5
C2—C7—C8	123.1 (2)	O2—C14—H142	109.5
C6—C7—C8	118.7 (2)	O2—C14—H143	109.5
C7—C8—C9	120.2 (2)	H141—C14—H142	109.5
C7—C8—C13	122.56 (19)	H141—C14—H143	109.5
C9—C8—C13	117.3 (2)	H142—C14—H143	109.5
C8—C9—C10	122.1 (2)	C15—C16—H161	109.5
C9—C10—C11	119.2 (2)	C15—C16—H162	109.5
C10—C11—C12	120.7 (3)	C15—C16—H163	109.5
C11—C12—C13	119.9 (3)	H161—C16—H162	109.5
O2—C13—C8	115.4 (2)	H161—C16—H163	109.5
O2—C13—C12	123.7 (2)	H162—C16—H163	109.5
C8—C13—C12	120.9 (2)	C15—C17—H171	109.5
N1—C15—C16	111.56 (19)	C15—C17—H172	109.5
N1—C15—C17	107.7 (2)	C15—C17—H173	109.5
N1—C15—C18	105.5 (2)	H171—C17—H172	109.5
C16—C15—C17	112.1 (2)	H171—C17—H173	109.5
C16—C15—C18	109.6 (2)	H172—C17—H173	109.5
C17—C15—C18	110.1 (2)	C15—C18—H181	109.5
N1—C1—H1	116.9	C15—C18—H182	109.5
C2—C1—H1	116.9	C15—C18—H183	109.5
C2—C3—H3	119.3	H181—C18—H182	109.5
C4—C3—H3	119.3	H181—C18—H183	109.5
C3—C4—H4	120.4	H182—C18—H183	109.5
C5—C4—H4	120.4		
			
C14—O2—C13—C8	174.2 (2)	C3—C4—C5—C6	0.9 (4)
C14—O2—C13—C12	−7.4 (4)	C4—C5—C6—C7	−0.1 (3)
O1—N1—C1—C2	−3.3 (4)	C5—C6—C7—C2	−0.9 (4)
O1—N1—C15—C16	−179.7 (2)	C5—C6—C7—C8	176.3 (2)
O1—N1—C15—C17	−56.2 (3)	C2—C7—C8—C9	121.0 (2)
O1—N1—C15—C18	61.4 (2)	C2—C7—C8—C13	−60.5 (4)
C1—N1—C15—C16	−0.3 (4)	C6—C7—C8—C9	−56.0 (3)
C1—N1—C15—C17	123.2 (2)	C6—C7—C8—C13	122.5 (2)
C1—N1—C15—C18	−119.2 (2)	C7—C8—C9—C10	179.5 (2)
C15—N1—C1—C2	177.4 (2)	C7—C8—C13—O2	−0.6 (4)
N1—C1—C2—C3	−26.8 (4)	C7—C8—C13—C12	−179.0 (2)
N1—C1—C2—C7	157.7 (2)	C9—C8—C13—O2	178.0 (2)
C1—C2—C3—C4	−175.7 (2)	C9—C8—C13—C12	−0.5 (4)
C1—C2—C7—C6	176.6 (2)	C13—C8—C9—C10	0.9 (4)
C1—C2—C7—C8	−0.5 (3)	C8—C9—C10—C11	−0.5 (5)
C3—C2—C7—C6	1.0 (3)	C9—C10—C11—C12	−0.2 (4)
C3—C2—C7—C8	−176.1 (2)	C10—C11—C12—C13	0.6 (5)
C7—C2—C3—C4	−0.2 (3)	C11—C12—C13—O2	−178.5 (2)
C2—C3—C4—C5	−0.8 (4)	C11—C12—C13—C8	−0.2 (4)

### Hydrogen-bond geometry (Å, °)

**Table:**

*D*—H···*A*	*D*—H	H···*A*	*D*···*A*	*D*—H···*A*
C3—H3···O1	0.93	2.26	2.806 (3)	117
C17—H171···O1	0.96	2.41	2.791 (3)	104
C18—H181···O1^i^	0.96	2.50	3.280 (3)	139

Symmetry codes: (i) −*x*+2, *y*−1/2, −*z*+2.
